# Effects of Chronic Calorie Restriction or Dietary Resveratrol Supplementation on Insulin Sensitivity Markers in a Primate, *Microcebus murinus*

**DOI:** 10.1371/journal.pone.0034289

**Published:** 2012-03-30

**Authors:** Julia Marchal, Stéphane Blanc, Jacques Epelbaum, Fabienne Aujard, Fabien Pifferi

**Affiliations:** 1 Mécanismes Adaptatifs et Evolution, UMR 7179 Centre National de la Recherche Scientifique, Muséum National d'Histoire Naturelle, Brunoy, France; 2 Institut Pluridisciplinaire Hubert Curien, Département d'Ecologie, Physiologie, Ethologie UMR 7178 CNRS Université Louis Pasteur, Strasbourg, France; 3 Centre de Psychiatrie et Neuroscience, UMR 894 Inserm, Faculté de Médecine, Université Paris Descartes, Paris, France; The University of Manchester, United Kingdom

## Abstract

The prevalence of diabetes and hyperinsulinemia increases with age, inducing metabolic failure and limiting lifespan. Calorie restriction (CR) without malnutrition delays the aging process, but its long-term application to humans seems difficult. Resveratrol (RSV), a dietary polyphenol, appears to be a promising CR mimetic that can be easily administered in humans. In this work, we hypothesized that both CR and RSV impact insulin sensitivity in a non-human primate compared to standard-fed control (CTL) animals. Four- to five-year-old male grey mouse lemurs (*Microcebus murinus*) were assigned to three dietary groups: a CTL group, a CR group receiving 30% fewer calories than the CTL and a RSV group receiving the CTL diet supplemented with RSV (200 mg·day^−1^·kg^−1^). Insulin sensitivity and glycemia were assessed using an oral glucose tolerance test (OGTT) and the homeostasis model assessment of insulin resistance (HOMA-IR index) evaluation after 21 or 33 months of chronic treatment. Resting metabolic rate was also measured to assess the potential relationships between this energy expenditure parameter and insulin sensitivity markers. No differences were found after a 21-month period of treatment, except for lower glucose levels 30 min after glucose loading in CR animals. After 33 months, CR and RSV decreased glycemia after the oral glucose loading without decreasing fasting blood insulin. A general effect of treatment was observed on the HOMA-IR index, with an 81% reduction in CR animals and 53% in RSV animals after 33 months of treatment compared to CTL. Chronic CR and dietary supplementation with RSV affected insulin sensitivity by improving the glucose tolerance of animals without disturbing their baseline insulin secretion. These results suggest that both CR and RSV have beneficial effects on metabolic alterations, although these effects are different in amplitude between the two anti-aging treatments and potentially rely on different metabolic changes.

## Introduction

Calorie restriction (CR) is the only non-genetic intervention that extends lifespan and delays the onset or reduces the emergence of age-associated diseases in short-lived species, such as invertebrates and rodents [1]. Ample evidence demonstrates the impact of dietary CR on lifespan extension and on metabolic diseases [2], [3] but the mechanisms by which CR exerts its anti-aging effects are not yet clear. Although the precise molecular mechanisms for this action remain controversial, some studies support the central importance of drastic modifications in energy metabolism [4]. Indeed, the mechanisms triggered by CR play a key role in the response to reduced energy availability, resulting in the induction of an altered metabolic state that is thought to promote longevity [5]. Insulin sensitivity is a relevant marker in evaluating the long-term health benefits of nutritional interventions, such as CR, as it increases systemic insulin sensitivity in rodents [6], [7], [8]. CR also reduces glycemia and the tissue accumulation of advanced glycation end products in rats [9]. In agreement with data from rodents, consistent metabolic changes are observed secondary to a CR treatment in the non-human primate *Macaca mulatta*, suggesting that the group with greater circulating insulin exhibits a faster age-dependent decline in survival rate [10], [11], [12]. In one study, CR monkeys (>18 years old) exhibit less diabetes and cardiovascular disease risks compared to control animals ([13]). Moreover, type II diabetes was not diagnosed in CR primates, in contrast to CTL groups, in which almost 50% presented diabetic or pre-diabetic states [14]. In human subjects, observations after 6 months of CR are consistent with those in monkeys and rodents in terms of lower insulin levels, in addition to basal metabolic rate and body temperature [15]. These data provide evidence that CR may have beneficial effects in protecting against diabetes or insulin resistance.

Nevertheless, due to social, economic and medical reasons, compliance to a CR paradigm seems impractical in humans, especially in the long term. Thus, recent research has developed CR mimetics [16], [17]. Among these, resveratrol (3,5,40-trihydroxy-trans-stilbene, RSV) has received special attention, as this polyphenol reduces many consequences of a high-calorie diet in mice and increases their survival by protecting them against insulin resistance [18]. Moreover, RSV inhibits insulin secretion of the pancreatic islets in normal rats [19], and similarly to CR, it lowers blood insulin in rodents [18], [20]. Thus, RSV may play an important role in energy regulation processes by targeting the insulin pathway. However, despite the numerous studies in short-lived species (flies, nematodes, mice and rats) on CR and aging, only a few have concerned long-lived species, and the effects of CR and those of a CR mimetic have not been compared in primates.

Thus, the objective of this study was to determine whether long-term RSV treatment prevents the age-related decrease in insulin sensitivity similarly to long-term CR in a primate species. This study occurred within the RESTRIKAL program [21], started in 2008, which aimed to investigate the long-term effects of CR or dietary RSV supplementation on physiological and behavioral parameters throughout the aging process of the grey mouse lemur (*Microcebus murinus*), which is a unique animal model to investigate aging (see [22] for review). Indeed longitudinal studies in this species can be performed on a longer range than those conducted in rodents, as the mouse lemur presents a median survival time of 5.7 years for males and an average lifespan of 11.0±0.2 years, based on data from the Brunoy breeding colony [22], living up to two-three times longer than mammals of equivalent body mass [23].

In this study, we chose to use the oral glucose tolerance test (OGTT) to measure insulin sensitivity. Even though the glucose clamp technique [24] is widely accepted as the standard reference for directly determining metabolic insulin sensitivity in humans, this method requires anesthesia, which is known to influence glycemia even in a fasting state. Additionally, OGTT reflects the efficiency with which the organism disposes of glucose after an oral glucose load. Thus, it provides useful information about glucose tolerance, and the OGTT data allow for the assessment of one of the surrogate validated indices of insulin sensitivity, the homeostasis model assessment (HOMA-IR index). In addition, the grey mouse lemur does not allow for large blood volume sampling because it has a low circulating blood volume. Thus, OGTT was the most suitable method to evaluate the glucose tolerance and insulin resistance of this primate model. Markers of insulin sensitivity were assessed through OGTT and the HOMA-IR index in two different groups of mouse lemurs: one group after 21 months of chronic CR or RSV treatment and another group after 33 months of chronic CR or RSV treatment. The resting metabolic rate (RMR) was also assessed, as it is a relevant component of daily energy expenditure estimation and a widely used predictive factor of body mass gain in human studies [25]. RMR has also been associated with metabolic syndrome in humans [26]. We were therefore interested in evaluating the relationships between this parameter and markers of insulin sensitivity.

## Materials and Methods

### Ethics statement

All experiments were performed in accordance with the Principles of Laboratory Animal Care (National Institutes of Health publication 86-23, revised 1985) and the European Communities Council Directive (86/609/EEC). The research was conducted under the authorization number 91–305 from the “Direction Départementale des Services Vétérinaires de l'Essonne” and the Internal Review Board of the UMR 7179. In accordance with the recommendations of the Weatherall report, “The use of non-human primates in research”, special attention was paid to the welfare of the animals during this work to minimize nociception [27].

### Animals and husbandry

All grey mouse lemurs studied were males that were born in the laboratory breeding colony of Brunoy (UMR 7179 CNRS/MNHN, France; European Institutions Agreement # 962773). These animals were part of the RESTRIKAL study [21]. The general conditions of captivity were constant. Animals were exposed to ambient room temperature (24–26°C) and relative humidity (55%). In captivity, seasonal variations of physiological and behavioral functions were entrained by alternating between 6 months of a long-day (LD) photoperiod (14∶10 light∶dark) and 6 months of a short-day photoperiod (SD) (10∶14 light∶dark), under artificial light (white light, 250 lux, wavelength peak at 488 nm). Animals were studied during the LD period, and measurements were made at least 2 months after the onset of the season, when their physiological status was stabilized. To minimize social influences, animals were housed individually in cages (50×40×30 cm), visually separated from each other. All tests were performed during the last 4 hours of the light phase, when animals begin their daily activity. The body mass of each animal was measured every week and the day before measurements to avoid stress from frequent handling. For ethical reasons, special attention was paid to the body mass evolution of the CR group because of their leanness during the LD period.

### Dietary interventions

Animals were fed fresh fruits and a daily mixture made up of ginger bread, cereals, milk and eggs [21]. Water was given *ad libitum*. Thirty-four animals were used in this study, randomly distributed into three different dietary groups at the age of 38±1 months: an *ad libitum* CTL group, a group of animals submitted to CR that were fed the same diet but received 30% less than CTL and a third group of animals supplemented with RSV that were fed the same quantity of food as CTL but supplemented with 200 mg of RSV per kilogram body mass per day (Sequoia Research Products, United Kingdom). This dosage was selected from the literature from studies in rodents, and was intermediate between the 5.2 mg/kg.d−1 of Baur et al. [28] and the 400 mg/kg.d−1 of Lagouge et al. [29]. To measure the exact quantity of food ingested by the animals, daily leftovers were measured and corrected for water evaporation. In this cross-sectional study, measurements were performed on two cohorts of animals: one cohort exposed to 21 months of treatment (n CTL = 5, n CR = 5, n RSV = 6, age: 59.9±1.2 months) and one cohort exposed to 33 months of treatment (n CTL = 5, n CR = 5, n RSV = 8, age: 71.8±1.1 months).

### Evaluation of fasting glycemia and glycemic response to OGTT

Glucose metabolism was assessed during the LD period, three months after the onset of the LD season. On the eve of the oral glucose tolerance test (OGTT), the food portion (fresh fruit and mixture) was removed from the cage. Fasting glycemia was measured using a non-invasive method with a hand-held blood glucose meter (Accu-Chek Active®, Hoffmann-La Roche, Switzerland) at the end of the resting phase of the animal but before food became available. OGTT was performed using a 1.75 g glucose.kg^−1^ body mass glucose challenge, consistent with previous research in rodents [30]. Animals received the oral glucose load between 1100 and 1115 am in the form of anhydrous glucose (Glucose Rectapur®, BDH Prolabo, UK) diluted in 0.6 ml of water, administered *per os* over 1 min. The hand-held glucometer required only 5 µl of total blood for each test stick, which were collected via the saphenous vein, in duplicate. Time points were determined on the basis of the results of a pilot study (Figure S1) in a sub-cohort of animals, with a lower sample size (CTL n = 3; CR n = 2; RSV n = 3). It showed no peak at 15 min after the oral load of glucose in the same experimental conditions as the final study. On the basis of the pilot results and since it is difficult to collect a high number and volume of blood samples in the grey mouse lemurs, we decided to focus on the most informative time points. Thus, blood was sampled at 30, 60 and 120 minutes after the glucose challenge. The first measurement corresponded to fasting glycemia.

### Plasma insulin assays

Fasting blood sampling for insulin assays was performed before the OGTT and at the same time as the fasting blood sampling for baseline glycemia. Blood samples were taken via the saphenous vein, without anesthesia, at the end of the resting phase but before food became available. Blood samples (100 to 150 µL) were collected in tubes containing EDTA and represented less than 1% of the blood volume of each animal. However, we failed to take a blood sample from two CR animals; thus, only 8 CR animals were available for insulin assessment. Blood samples were centrifuged at 2000 *g* at 4°C for 30 min. Before storage of plasma at −80°C, the samples underwent a second centrifugation at 2000 *g* at 4°C for 10 min. Plasma insulin was measured in simplicate due to the minimal plasma volume collected using the Human Insulin assay method (Elisa technology, Cat. # EZHI-14K).

### Insulin resistance index

Basal insulin resistance was estimated by HOMA-IR, which was calculated using fasting glucose and fasting insulin as an assessment of basal insulin resistance [31]. Fasting insulin and glucose collected at the time of the OGTT were used in the HOMA-IR formula as follows: HOMA-IR = Fasting insulin (µIU/mL) * Fasting glucose (mmol/L)/22.5 [32].

### Resting metabolic rate

Oxygen consumption was measured with a closed-circuit respirometer one month after the OGTT and the fasting insulin level assays (i.e., after 22 or 34 months of treatment). For this nocturnal species, RMR measurements were performed during their daily resting period, 4–6 h after the beginning of the light period, to avoid torpor metabolism. Animals were trained to nest in the respiratory chamber, which consisted of 2.5-L opaque chamber with a woven floor to absorb any urine. During the experiment, the respiratory chamber was placed in a cabinet at a controlled ambient temperature of 25.0±0.5°C, a value within the thermo-neutral zone defined for the mouse lemur [33]. After a 20 min habituation phase under constant airflow ventilation (2 L·min^−1^) drawn through the respirometry chamber from bottom to top, the chamber was closed for 40 min. The VO_2_ consumed by the animal was calculated from the initial and final concentrations of O_2_ in the chamber, which were measured on dried gas using a Servomex 570 A paramagnetic gas analyzer (accuracy 0.01% O_2_). The analyzer was routinely calibrated with N_2_ and atmospheric air. O_2_ consumption was expressed as mL O_2_·h^−1^ and was adjusted for the body mass of the animal [34].

### Data analysis and statistics

The Shapiro-Wilk goodness-of-fit test was applied to determine whether the sample data were likely to derive from a normally distributed population. All data were analyzed to estimate and test the individual and interactive effects of treatments (diets) and treatment duration using two-way ANOVA. Bonferroni post-hoc tests were used to determine the significance of differences between each pair of the three groups. Two-way ANOVA for repeated measures with consecutive multiple comparison tests was used to compare the time courses of glucose during OGTT among the CTL, CR and RSV animals. Simple linear regressions were also used to determine the significance levels of correlations among variables. All analyses were performed with SYSTAT version 12 (SYSTAT Software, Inc., San Jose, California). A level of p<0.05 was set for significance, and all values are expressed as means ± standard errors of the mean (SEM).

## Results

### Effects of age and treatments on body mass and OGTT-related parameters

**Figure 1:**
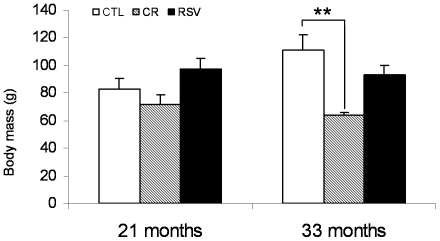
Body mass at oral glucose tolerance test in control (CTL), calorie restricted (CR) and resveratrol supplemented (RSV) animals after 21 months of treatment (n CTL = 5, n CR = 5, n RSV = 6) and after 33 months of treatment (n CTL = 5, n CR = 5, n RSV = 8). Data are expressed as means±SEM. Statistical significance (**) p<0.01 when comparing CTL and CR animals.

As shown in Figure 1, body mass varied significantly among the three groups, as we observed an important effect of treatment (dF = 2/28, F = 8.562, p = 0.001), while no general effect of age occurred (dF = 1/28, F = 0.719, p = 0.404). However, body mass was not different among the three groups after 21 months of treatment; only the CR animals presented significantly lower body mass after 33 months of treatment compared to the CTL animals (p = 0.003).

**Figure 2:**
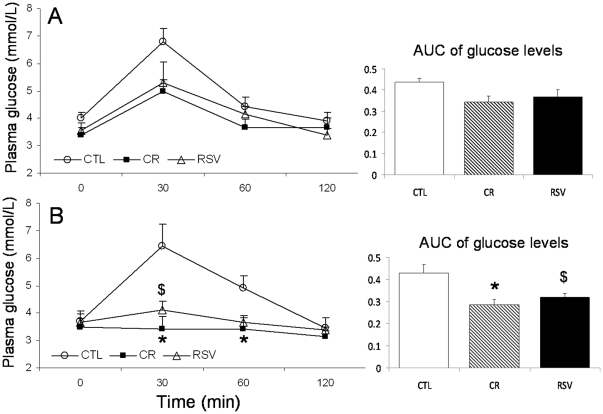
Plasma glucose concentrations and area under curves (AUC) during oral glucose tolerance test in control (CTL), calorie restricted (CR) and resveratrol supplemented (RSV) animals. A: after 21 months of treatment and B: after 33 months of treatment. Data are expressed as means±SEM. Statistical significance (*) p<0.05 when comparing CTL and CR animals, ($) p<0.05 when comparing CTL and RSV animals.

The OGTT results are presented in Figure 2, for each treatment and by age group. OGTT measurements significantly varied according to the treatment (dF = 2/28, F = 6.926, p = 0.004), regardless of the duration of the treatment (dF = 1/28, F = 2.418, p = 0.131). Additionally, OGTT values varied within the time course (dF = 3/84, F = 39.68, p<0.001) according to the treatment (6/84, F = 5.74, p<0.001) and treatment duration (dF = 3/84, F = 3.61, p = 0.017). After 21 months of treatment, the CR and RSV animals presented slight decreases in glucose compared to the CTL animals, but neither was significant. However, after 33 months of treatment, post-hoc tests revealed that the CR animals presented lower plasma glucose levels at 30 and 60 minutes after the beginning of the OGTT (p = 0.012 and p = 0.041, respectively). The RSV animals presented lower glucose levels at 30 minutes after glucose loading (p = 0.043), while no significant difference was found 60 minutes after the glucose loading (p = 0.074). Additionally, baseline glucose levels were not different among the three groups at 21 or 33 months nor were glucose levels 120 minutes after the glucose loading.

Areas under the curve (AUCs) varied significantly with treatment, as we found a general effect (dF = 2/28, F = 9.073, p = 0.001) regardless of the duration of the treatment (dF = 1/28, F = 2.979, p = 0.095). Despite the lack of a significant difference in AUC between either of the treated groups and the CTL group after 21 months of treatment (CR: p = 0.263 and RSV: p = 0.543), the AUC of the CR animals was lower after 33 months (p = 0.017), as was the AUC of the RSV animals (p = 0.048) compared to the CTL group.

### Effects of age and treatments on fasting plasma insulin, HOMA-IR and RMR

A nearly significant effect of treatment on fasting insulin was revealed (dF = 2/26, F = 3.141, p = 0.06), while no significant effect of age was found (dF = 1/26, F = 1.722, p = 0.201), nor was a crossed effect found (dF = 2/26, F = 1.856, p = 0.176) (Table 1).

**Table 1:** Sample size, fasting plasma levels of insulin and HOMA-IR obtained during the OGTT after 21 months of treatment or after 33 months of treatment.

	21 months of treatment			33 months of treatment		
	CTL	CR	RSV	CTL	CR	RSV
**Fasting insulin (µIU/mL)**	31.35±13.40 n = 5	12.74±4.09 n = 4	38.04±15.80 n = 6	86.85±26.73 n = 5	14.47±2.89 n = 4	35.71±14.95 n = 8
**HOMA-IR**	5.72±2.44 n = 5	1.72±0.40 n = 4	5.96±2.25 n = 6	12.85±2.89 n = 5	2.46±0.69 n = 4	6.05±2.65 n = 8
**Resting metabolic rate (mL O2/h/g^−0.67^)**	3.51±0.30 n = 2	3.45±0.15 n = 5	3.61±0.37 n = 6	2.74±0.32 n = 4	3.58±0.81 n = 5	4.75±0.33 n = 7

Data are expressed as means±SEM. Control (CTL), calorie restricted (CR), resveratrol (RSV).

HOMA-IR varied according to the treatment (dF = 2/26, F = 3.368, p = 0.048), regardless of the duration of the treatment (dF = 1/26, F = 1.555, p = 0.224). The crossed effect between treatment and age was not significant (dF = 2/26, F = 1.167, p = 0.327). HOMA-IR in the CR animals was lower than in CTL by 70% after 21 months of treatment and by 81% after 33 months of treatment. In the RSV animals, HOMA-IR was similar to the HOMA-IR of CTL mouse lemurs after 21 months of treatment but was attenuated by 53% after 33 months of treatment compared to the CTL animals (Table 1).

RMR did not vary with treatment across the two ages (dF = 2/23, F = 2.522, p = 0.111). Similarly, no effect of age was found (dF = 1/23, F = 0.184, p = 0.678), nor was a crossed effect found (dF = 2/23, F = 1.89, p = 0.185) (Table 1).

### Relationships between insulin sensitivity parameters and metabolic parameters

We combined the data from both cohorts for the simple linear regressions (Table 2). Fasting insulin and body mass were highly related in the CTL animals (r = 0.89, p<0.001). However, this relation was not observed for the CR (r = 0.122, p = 0.773) or RSV animals (r = 0.122, p = 0.679). Similarly, HOMA-IR and body mass were related in the CTL animals (r = 0.943, p<0.001) but not in the CR (r = 0.283, p = 0.496) or RSV animals (r = 0.077, p = 0.794). In addition, the CTL animals exhibited a strong positive relation between HOMA-IR and RMR (r = 0.847, p = 0.033), which was not the case for the CR (r = 0.346, p = 0.401) or RSV animals (r = 0.077, p = 0.802).

**Table 2:** Table of simple linear regressions in CTL, CR and RSV animals.

Dependent variables	Independent variables	CTL group	CR group	RSV group
**HOMA-IR**	**Body mass (g)**	n = 10 r = 0.940 ***	n = 8 r = 0.122 NS	n = 14 r = 0.122 NS
**Fasting insulin (µIU/mL)**	**Body mass (g)**	n = 10 r = 0.943 ***	n = 8 r = 0.283 NS	n = 14 r = 0.077 NS
**HOMA-IR**	**RMR (mL O2/h/g-0.67)**	n = 6 r = 0.847 *	n = 8 r = 0.346 NS	n = 13 r = 0.077 NS

Sample size for each diet groups and Pearson correlation coefficient r are given for each regression, as dependent and independent variables. Statistical significance was considered when p<0.05 (*), p<0.001 (***), NS: non significance. Control (CTL), calorie restricted (CR), resveratrol (RSV).

## Discussion

This study provides insight into whether the effects of CR or RSV on insulin and glucose functions can occur in the grey mouse lemur at early–middle age. Our results indicate that beneficial effects of CR and RSV appeared after 33 months by decreasing glycemia after a glucose challenge. Additionally, chronic CR and RSV treatments led to enhanced insulin sensitivity, as indicated by the HOMA-IR index values, compared to the CTL group, whatever the duration of treatment.

In humans, the decline of gluco-regulatory functions and insulin resistance occur in the great majority of elderly, with the states of glucose intolerance due in part to a decline in peripheral tissue sensitivity to insulin [35]. To maintain glucose homeostasis, the compensatory response to a decrease in insulin-stimulated glucose uptake is an increase in plasma insulin concentration, and several reports have documented a significant direct relationship between the magnitude of insulin resistance and the degree of associated hyper-insulinemia in non-diabetic individuals (see [36] for a review). Nevertheless, the capacity of insulin to stimulate glucose uptake can vary substantially among non-obese persons with no apparent disease [37]. During the OGTT, our results did not reveal an effect of age on the glycemia response to the glucose challenge, neither on fasting insulin nor on HOMA-IR. Nevertheless, it is important to note that the study was conducted on 5- to 6-year-old animals, corresponding to middle age in this species [22].

In CTL animals, both fasting insulin and HOMA-IR were strongly positively correlated with body mass. To exclude any cohort effect, we compared body weights of the two cohorts of animals after the same time of treatment and found no significant differences (data not shown). These findings confirm previous research in humans that showed a positive correlation among body mass index, plasma insulin and plasma glucose in both normal and obese populations [38]. Their study revealed that total body mass may be a good indicator of the need to screen for metabolic disorders, such as type II diabetes, in adult subjects. However, in most human studies that measured fasting insulin, the study samples were not representative of the normal population because they included a large proportion of overweight subjects [39], [40]. Similarly, in the majority of rodent studies, the animals were overweight [41], genetically obese [42] or on a high-calorie diet [43], [44], which induce both glucose and insulin metabolism disorders and disturbances of *in vivo* insulin action [45]. Therefore, the correlations identified in the CTL mouse lemurs in this study support the relationships among body mass gain, insulin resistance and the decline of gluco-regulatory functions, all of which may occur even in a normal population without nutritional modification. Moreover, the negative correlation between the HOMA-IR and RMR in the CTL animals supports the hypothesis that glucose metabolism disorders and pre-diabetic states might be characterized by a lower energy expenditure, which facilitates body mass gain. One four-year follow-up study in human subjects indicated that individuals with metabolic syndrome exhibited a significantly lower RMR adjusted for fat-free mass compared to the control group, indicating that a low rate of energy expenditure is a risk factor for body mass gain and obesity [25].

One of the main objectives of this work was to test whether CR may improve insulin sensitivity in the mouse lemur. We demonstrated that chronic CR started at the adult age improved markers of insulin sensitivity. After 33 months of treatment, CR induced a decrease in body mass when compared to CTL. Weight loss produces numerous benefits in altered metabolic states, including reduced glycemia, reduced fasting insulin and increased insulin sensitivity [46]. Nevertheless, when human subjects returned to a normal diet after a 24-h reduction in food energy, the blood glucose concentration increased, suggesting that the major underlying factor is the reduction in calorie intake rather than the body mass loss [47]. In mouse lemurs, CR reduced the glycemia response to a glucose challenge (OGTT) after 33 months of treatment, but some studies have revealed even faster CR effects. In over-fed rodents, a moderate CR for 48 h causes changes in the liver and skeletal muscle metabolism by lowering the glucose production rate [48]. Similarly, an acute but short-term CR without malnutrition has been effective in the care of type II diabetes by improving insulin-stimulated glucose uptake in humans [49]. However, the CR mouse lemurs in this study were not overweight or previously over-fed, suggesting that long-term CR has benefits in non-diabetic individuals by decreasing the glycemia threshold after the glucose challenge by inducing metabolic disturbances that only occur with long-term treatment.

We did not identify any correlations between the markers of insulin sensitivity and body mass or RMR in CR animals. In obese humans, a strong negative correlation exists between weight loss and insulin sensitivity that is statistically superior to the correlations between weight loss and fasting glucose, fasting insulin and HOMA-IR [50]. Similarly, insulin sensitivity can improve because of weight reduction in obese individuals on very low-calorie diets [51]. Here, in CR mouse lemurs, the absence of a relationship among the studied parameters indicates a non-dependence between the decrease of glucose level after the glucose challenge and the weight loss. This finding suggests that the enhancement of insulin sensitivity was not only due to weight loss but also to metabolic changes that occurred during the chronic CR. In addition, despite no variation with treatment in RMR, the lack of a relationship between this parameter and the estimation of insulin sensitivity indicates that modifications in glucose or hormonal metabolism were independent of metabolic status in CR animals. However, in future investigations, we should test the relationships between the variations in body mass over the duration of treatment and the glucose or insulin parameters, and the animals could be returned to a normal diet to assess whether the body mass loss or the reduction in energy expenditure is responsible for the improved insulin sensitivity.

The second aim of this study was to investigate whether the beneficial effects observed in CR animals would be replicated in RSV animals, as the most recent data from animal studies present a promising perspective of the potential use of RSV in preventing and treating metabolic disorders, such as diabetes and obesity [29]. Primarily, we did not observe any difference between the body masses of the RSV and CTL animals, in contrast to what we observed between the CR and CTL animals after 33 months of treatment. This finding agrees with previous results from the one-year report of the RESTRIKAL study [21]. Meanwhile, RSV was reported to reduce body mass gain in adult mouse lemurs after one month of treatment [52] or in rats fed a high-fat diet [53], but its effects are controversial because such results have not been replicated in other studies [54]. One reason for these discrepancies is certainly the short-term treatments that have been used, during which organisms presented decreased body mass that occurs because of an increase in total energy expenditure [21]. As shown by Dal-Pan et al., RSV produced an activation of metabolism without a loss in body mass, in contrast to CR animals, which exhibited a reduction in energy expenditure, particularly during the LD season, when the energy constraints are more important than in the short-day season. In this study, the body masses of the RSV mouse lemurs did not differ from the CTL mouse lemurs and did not correlate with plasma insulin or with HOMA-IR, in contrast to what we observed for the CTL animals. As shown in the CR animals, the enhancement of insulin sensitivity markers in the RSV-treated animals was not related to changes in body mass, even though, as opposed to the CR animals, they had the same amount of daily calorie intake as the CTL animals. Indeed, the RSV animals exhibited lower blood glucose levels during OGTT after 3 years of treatment. RSV and other wine polyphenols have been proposed to affect blood glycemia through the inhibition of glucose absorption in the intestine or of its uptake by peripheral tissues [55]. In addition, RSV decreases insulin secretion and delays the onset of insulin resistance, possibly by inhibiting ATP- and voltage-dependent channels in pancreatic beta-cells [56]. We did not observe lower fasting insulin levels in the RSV animals, despite a slight decreasing tendency compared to the CTL mouse lemurs. Similar findings have been shown in type 2 diabetic patients, in whom a positive effect on glycemia was clearly revealed after grape seed extract ingestion, but this did not result in statistically significant changes in blood insulin levels [55]. Data from the literature and the results of this study indicate that the capacity of RSV to induce changes in baseline blood insulin levels remains controversial. Our inconclusive findings could have been due to the dose given to the animals (200 mg·day^−1^·kg^−1^), as many publications reveal a dose-dependent activity of this molecule [20]. We can also suggest that RSV may act on glucose metabolism by decreasing the glycemia response during an OGTT, which may be due to a physiological insulin-secretory response to glucose. However, we only assessed the basal insulin level, and we cannot draw conclusions about the evolution of insulin release in response to the glucose challenge. However, Szkudelski demonstrated that RSV decreases the insulin secretion of rat pancreatic islets when incubated with different glucose concentrations, while the baseline level of insulin was unchanged [19]. Moreover, it was clearly observed that a 50 mg·kg^−1^ dose of RSV decreased blood insulin without diminishing blood glucose, supporting the direct insulin-suppressive action of RSV in vivo and in vitro [57].

In conclusion, we demonstrated that CR and RSV decreased glycemia during an OGTT without disturbing the fasting insulin or fasting glucose levels. The fact that glucose decreased in response to a glucose challenge without concomitant changes in fasting insulin levels means that CR and RSV were efficient in enhancing the insulin sensitivity of our animals. These effects were revealed after 33 months of chronic treatment, when the animals were adults; thus, these results are promising and have encouraged us to investigate the impact of these nutritional interventions in old mouse lemurs. The two anti-aging treatments had effects of different amplitudes that potentially rely on different metabolic changes, which suggests that different pathways are involved in their respective responses. One of the hypothetically similar pathways between CR and RSV supplementation involves SIRT1, which we plan to assess in the context of CR and RSV in future investigations. The beneficial effect of RSV appears to be mediated by proteins also involved in CR pathways, such as AMP kinase and PGC-1α, but the precise mechanisms are still unclear [29], [17]. However, lowering energy intake by CR or by CR mimetic drugs, such as RSV, protects organisms against diseases and metabolic dysfunctions, in part by hormesis mechanisms that improve cellular stress resistance [58]. Therefore, the possibility of following numerous physiological parameters longitudinally under controlled conditions offers potential value in the context of clarifying the characteristics and kinetics of the effects of CR and RSV on insulin sensitivity, especially because of their potential recommendations for diabetes prevention and care.

## Supporting Information

Figure S1 **Results** **of the pilot study.** (See the Materials & Methods section in the manuscript.) Plasma glucose concentrations during oral glucose tolerance test in control (CTL; n = 3), calorie restricted (CR, n = 2) and resveratrol supplemented (RSV, n = 3) animals, after 21 months of treatment. There was no difference between the point at 15 minutes and the point at 30 minutes when considering each group (CTL 15 min vs 30 min, dF = 2; t = 0.051, p = 0.964; CR 15 min vs 30 min, dF = 1, t = −1.687, p = 0.341; RSV 15 min vs 30 min, dF = 2, t = −0.679, p = 0.567) Data are expressed as means±SEM.
(TIF)
