# Supplementary figures and images for: Effects of Chronic Calorie Restriction or Dietary Resveratrol Supplementation on Insulin Sensitivity Markers in a Primate, Microcebus murinus
Source: PLoS One. 2012 Mar 30;7(3):e34289. doi: 10.1371/journal.pone.0034289 (PMC3316613; doi:10.1371/journal.pone.0034289)

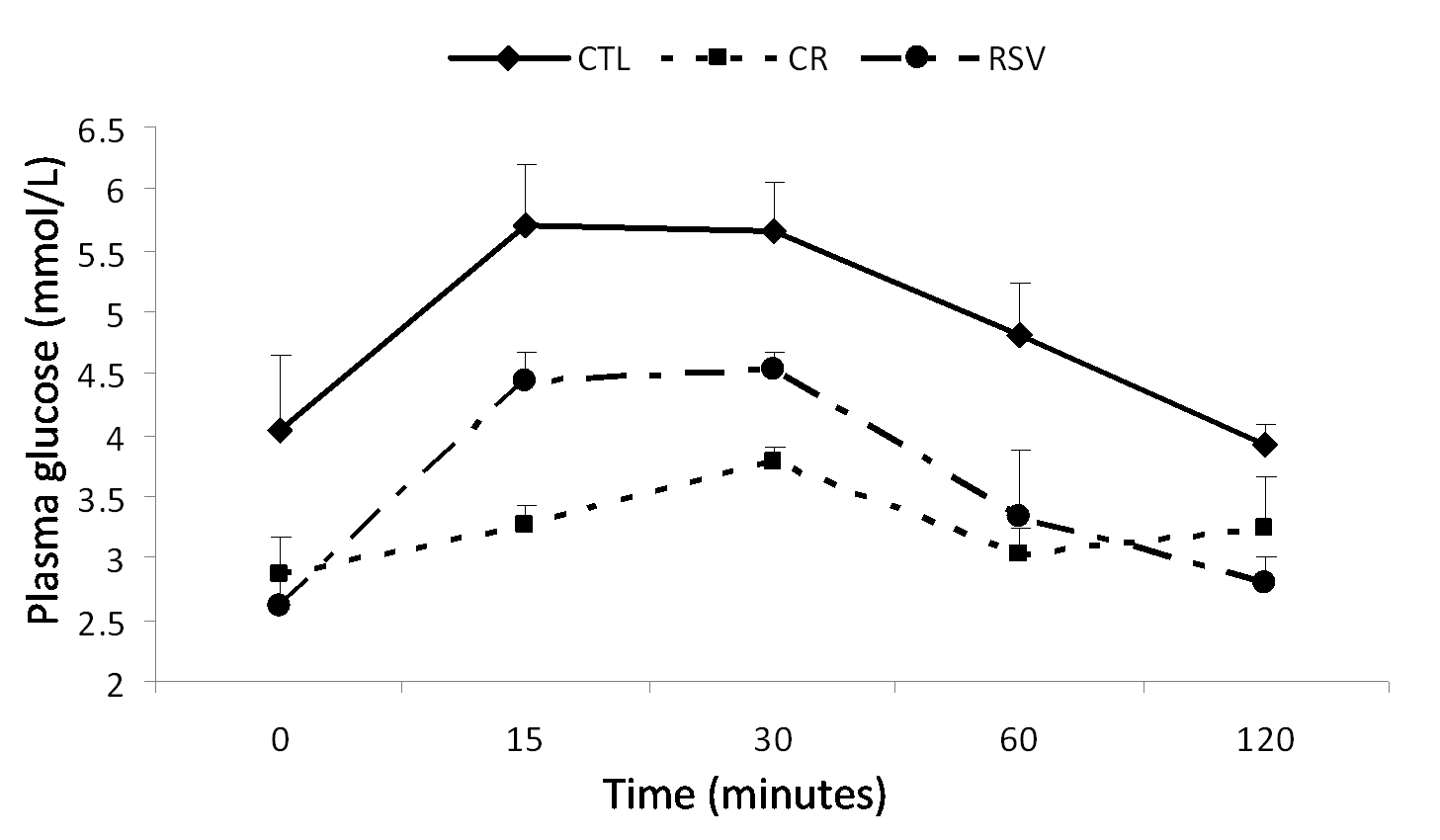

Supplement: Figure S1 — Results of the pilot study. (See the Materials & Methods section in the manuscript.) Plasma glucose concentrations during oral glucose tolerance test in control (CTL; n = 3), calorie restricted (CR, n = 2) and resveratrol supplemented (RSV, n = 3) animals, after 21 months of treatment. There was no difference between the point at 15 minutes and the point at 30 minutes when considering each group (CTL 15 min vs 30 min, dF = 2; t = 0.051, p = 0.964; CR 15 min vs 30 min, dF = 1, t = −1.687, p = 0.341; RSV 15 min vs 30 min, dF = 2, t = −0.679, p = 0.567) Data are expressed as means±SEM. (TIF) [file pone.0034289.s001.tif]
